# Elevated serum leptin levels in patients with acute myocardial infarction; correlation with coronary angiographic and echocardiographic findings

**DOI:** 10.1186/1756-0500-5-262

**Published:** 2012-05-29

**Authors:** Hadi AR Hadi Khafaji, Abdul Bari Bener, Nasser M Rizk, Jassim Al Suwaidi

**Affiliations:** 1 Heart Hospital, Hamad Medical Corporation, Doha, Qatar; 2 Dept. of Adult Cardiology, Dept. of Medical Statistics and Epidemiology, Doha, Qatar; 3 Biomedical Department, Qatar University, Doha, Qatar, Heart Hospital, Hamad Medical Corporation, Qatar, Biomedical Department, Qatar University, Doha, Qatar; 4 Department of Adult Cardiology Hamad Medical Corporation - Heart Hospital, P.O. Box 3050, Doha, Qatar

**Keywords:** Serum leptin, Acute myocardial infarction, Angiographic findings, Echocardiography

## Abstract

**Background:**

To assess the relationship between serial serum leptin levels in patients with acute myocardial infarction (AMI) who received thrombolysis and the degree of coronary atherosclerosis, coronary reperfusion, echocardiographic findings, and clinical outcome. 51 consecutive patients presenting with AMI were studied. Clinical characteristics including age, sex, body mass index (BMI) and cardiovascular risk factors were recorded. Serial serum leptin levels at the time of admission and subsequently at 0, 6, 12, 24, 36, 60 hours afterwards were obtained. Coronary angiography was performed in 34 patients; the relation between serum leptin levels and evidence of coronary reperfusion as well as the extent of coronary atherosclerosis according to the coronary artery surgery study classification (CASS) were evaluated. Echocardiographic evaluation was performed in all patients. 36 matched patients were enrolled as control group who had serum leptin level 9.4 ± 6.5 ng/ml.

**Results:**

The patients mean age was 50.5 ± 10.6 years. There were 47 males and 3 females. 37.1% were diabetics, 23.5% were hypertensive, 21.6% were dyslipidemic and 22.7% were obese (BMI ≥ 30). Leptin concentrations (ng/ml) increased and peaked at the 4th sample (36 hrs) after admission (mean ± SD) sample (1) =9.55 ± 7.4, sample (2) =12.9 ± 8.4, sample (3) =13.8 ± 10.4, sample (4) =18.9 ± 18.1, sample (5) =11.4 ± 6.5, sample (6) =10.8 ± 8.9 ng/ml. There was a significant correlation between serum leptin and BMI (r = 0.342; *p* = 0.03). Leptin levels correlated significantly to creatine kinase level on the second day (r = 0.43, *p* ≤ 0.01). Significant correlation of mean serum leptin with the ejection fraction (*P* < 0.05) was found. No difference in timing of peak serum leptin between patients who achieved coronary reperfusion vs. those who did not (*p* = 0.8). There was a trend for an increase in the mean serum leptin levels with increasing number of diseased vessels. There was no correlation between serum leptin levels and outcome neither during the hospitalization nor at 9 months follow up.

**Conclusion:**

Serum leptin levels increase after myocardial infarction. Serum leptin level may be a predictor of the left ventricular ejection fraction and the degree of atherosclerosis but not of coronary reperfusion.

## Background

Leptin is the 16,000 Dalton protein product of the obesity gene (ob) [1]. Leptin has a role in the body weight maintenance in humans. It is released into the blood stream, where it binds to leptin-binding protein and is transported into the cerebrospinal fluid [2-5] and exerts its major effect on the hypothalamus. The role of leptin in coronary artery vasoreactivity has been raised in a study by Sundell J et al. [6] in obese and non-obese patients, suggesting that leptin might have a role in the regulation of the myocardial blood flow. A preliminary study in a small number of patients suggested that leptin levels might increase with acute myocardial infarction [7]. Leptin is found to have multiple roles in the cardiovascular system. A number of investigators suggested its role as a vasoactive substance [7]. There is also some evidence that leptin may have a role in obesity-related hypertension [8]. In a human study, a correlation was seen between the serum concentration of leptin and blood pressure among patients with essential hypertension [9]. Leptin also may have a prothrombotic effect [10]. This effect appears to be mediated through the platelet leptin receptor. In a previous study we suggested that TNF alpha may represent a modulator of leptin action in the hypothalamus, such finding may have implication in the setting of acute myocardial infarction [11].

The aims of the current study were to evaluate serial levels of serum leptin among patients presenting with acute myocardial infarction (AMI) and whether there is a correlation between leptin and coronary reperfusion as well as with angiographic and echocardiographic data.

## Methods

### Study design

This study was conducted at Hamad General Hospital. 51 patients who were admitted to the Coronary Care Unit with a diagnosis of acute coronary syndrome (ACS) were studied; after written informed consent for participation in the study was obtained from participants. The definition of ACS in this study is according to the definition for acute myocardial infarction by the joint committee of American College of Cardiology/European Society of Cardiology [12]. Patients with major co-morbid conditions including renal failure were excluded from the study. Hamad Medical Corporation institutional review board approved the study.

Baseline clinical characteristics including age, sex, cardiovascular risk factors, and complete cardiovascular physical examination findings were recorded. The mode of administered therapy was also recorded. 49 patients with ST elevation myocardial infarction (STEMI) were given thrombolytic therapy with either metalyse, or streptokinase [13]. Tow patients suffered non-ST elevation myocardial infarction (NSTEMI). None of these patients developed acute complication such as cardiogenic shock or acute homodynamic disturbance. Height, weight, body mass index, smoking habit, alcohol intake, diabetes mellitus, hypertension, lipid profile including serum cholesterol, high density and low density lipoprotein, and serum creatinine of patients were analyzed. Measurements of serum creatinine kinase, creatinine kinase-MB portion, troponin T levels were taken at the time of admission (0 time) and subsequently at 6, 12, 24, 36, 60 hours afterwards. Full echocardiographic studies were performed for all patients on the 2^nd^ day of admission. Variables including left ventricular ejection fraction (LV EF%), left ventricular end-systolic dimension (LVESD), left ventricular end-diastolic dimension (LVEDD), left atrial size, right ventricular dimension (RVD) and right ventricular systolic pressure (RVSP) were all recorded. Coronary angiography was performed in 36 patients as a routine clinical check up. Extent of diseased vessels involved; whether single vessel, two -vessel, or three-vessel diseases, and whether re-canalization achieved (defined non-invasively as rapid resolution of ST segment elevation reperfusion arrhythmias and chest pain resolution after thrombolytic therapy or angiographically by the evidence of non-occluded culprit coronary artery) were recorded.

36 matched (age and BMI) stable cardiac patients with the same age and sex were taken as a control group and their data were collected from the cardiology out patient department.

### Biochemical analyses

Blood samples were collected in plain tubes from each patient at the time of admission and then at 6, 12, 24, 36, 48, 60 and 84 hours. All samples were separated and analyzed immediately with the exception of serum leptin. Aliquots for serum leptin were stored at −80 °C until the collection was completed. Leptin concentrations were measured by ELISA method. Linco Research, Inc USA supplied the reagents. The measuring range was 0.5–100 ng/ml. According to manufacturer the assay has high correlation with their radioimmunoassay for human leptin (r = 0.967), lower detection limit is 0.5 ng/ml and the concentration variations (CVs) at concentrations of 3.2 ng/ml & 8.18 ng/ml were 5% & 3.0% respectively [14]. Total cholesterol (TC), Triglycerides (TG) and HDL-cholesterol (HDL-C) were determined enzymatically by Hitachi 917, Roche, Mannheim, Germany. LDL- cholesterol (LDL-C) was estimated among those with TG concentrations < 4.5 mmol/l as TC - HDL-C - Tg/2.2 [15]. Those with TG >4.5 mmol/l were measured directly by reagents from the same manufacturer [16]. Between-run imprecision (CVs) for TC, TG & measured LDL- C were 3.0%, 4.0% 2.0% and 2.0% respectively. Troponin T & CKMB were measured immunochemically by Elecys 2010, Roche, according to the manufacturer protocols. Imprecision, between run (CVs), for troponin & CKMB were 2% & 3% respectively [17].

### Normal fasting level of serum leptin

Normal fasting ranges for serum leptin are directly correlated with the degree of adiposity, with BMI range 18–25; serum leptin level for men (3.8 ± 1.8 ng/ml), and for women (7.4 ± 3.7 ng/ml). Serum leptin level rises approximately 2.5 times faster in women per unit BMI as compared to man. Keeping in mind that a diurnal rhythm of serum leptin concentrations, the values being 20 to 40 percent higher in the middle of the night as compared with daytime [18,19]. The peak shifts are in parallel with shifts in the timing of meals [20].

### Coronary angiography and echocardiographic analysis

Coronary angiography was performed according to the standard Judkin technique femoral approach either on admission or during the recovery period. Several views of each coronary artery were analyzed. The severity of arterial stenosis (defined as maximal percent reduction in luminal diameter) was determined according to visual estimation using the coronary artery surgery study classification (CASS) study analysis. Significant coronary stenosis was defined as a 70% lumen narrowing or > than 50% lumen narrowing of the left main coronary artery. The extent of coronary artery disease was classified as 1, 2 or 3 vessels according to number of major coronary arteries with significant stenosis [21].

Transthoracic 2 dimensional echocardiography 3 megahertz, 5500 Hewlett Packard Sonos machine was used for the study. Images were captured in the 5 standard views, parasternal long axis views, short axis view and 4, 2 &3 chamber views according to criteria of American society of echocardiography. The measurement of left ventricular dimensions was performed from 2 dimensional targeted M-mode at end diastolic and end systolic dimensions and subsequently LV ejection fraction was calculated. Left atrial size at end systole was measured from the parasternal long axis view. Right ventricular dimensions were recorded from the parasternal long axis view. Conventional Doppler measurements were obtained using tricuspid inflow, mitral early and late diastolic inflow velocities, volumetric relaxation times were also recorded. Wall motion abnormalities were recorded using 16 segment models [22].

### Statistical analysis

The data were analyzed by using the Statistical Packages for Social Sciences [SPSS] version 19 [23]. Data were expressed as mean and standard deviation (SD) unless otherwise stated; student *t* test was used to ascertain the significance of difference between mean values of two continuous variables and confirmed by non-parametric Mann Whitney test. Fisher exact and Chi square test were performed to test for difference in proportions of categorical variable between two and more groups. The Pearson’s correlation coefficient was used to evaluate the strength association between two variables. The level of *P* < 0.05 was considered as the cut off value for significance. Repeated measures ANOVA utilized to test differences in serial leptin measures.

## Results

Baseline clinical characteristics of the studied patients shown in Table 1. The mean age of patients was 50.5 ± 10.6 years. There were 47 males and 4 females. 37.1% had diabetes mellitus, 23.5% had hypertension, 21.6% had high cholesterol, and 22.7% were obese (BMI ≥ 30). Thirty six matched (with age and BMI) stable cardiac patients were taken as a control group. The mean serum leptin level of the control group was 9.43 ± 6.5 ng/ml.

**Table 1:** Clinical baseline characteristics compared with mean and peak leptin sample

**Variables**		**Mean Leptin**		**Peak Leptin**	
		**Normoleptinemia**	**hyperleptinemia**	**Normoleptinemia**	**hyperleptinemia**
		**N = 13 (29.5%)**	**N = 31 (70.4%)**	**N = 5 (13.1%)**	**N = 33 (86.8%)**
Sex	Male	11(84.6%)	31(100.0%)	4(80.0%)	32 (97.0%)
	Female	2(15.4%)	0(0.0)	1(20.0%)	1(3.0%)
Age group	≤50 years	7(53.8%)	17(54.8%)	2(40.0%)	17 (51.5%)
	>50 years	6(46.2%)	14(45.2%)	3(60.0%)	16 (48.5%)
Diabetic	Yes	8(61.5%)	9(29.0%)	5(100.0%)	10 (30.3%)
	No	5(38.5%)	22(71.0%)	0(0.0)	23 (69.7%)
Hypertensive	Yes	4(30.8%)	5(16.1%)	1(20.0%)	6(18.2%)
	No	9(69.2%)	26(83.9%)	4(80.0%)	27(81.8 %)
Body mass index (Kg/m^2^)	<25	5(45.5%)	2(6.9%)	2(40.0%)	3(10.0%)
	25-30	4(36.4%)	19(65.5%)	3(60.0%)	18(60.0%)
	>30	2(18.2%)	8(27.6%)	0(0.0)	9(30.0%)
High Cholesterol >5.2 mmol/l	Yes	5(38.5%)	4(12.9%)	1(20.0%)	7(21.2%)
	No	8(61.5%)	27(87.1%)	4(80.0%)	26(78.8%)
HDL*	Abnormal (<1)mmolL	10(90.9%)	10(37.0%)	5(100.0%)	12(41.4%)
	Normal(>1) mmol/L	1(9.1%)	17(63.0%)	0(0.0)	17(58.6%)
LDL	≤3.3 mmol/L	4(40.0%)	20(69.0%)	3(75.0%)	19(61.3%)
	>3.3 mmol/L	6(60.0%)	9(31.0%)	1(25.0%)	12(38.7%)
Triglyceride	≤1.7 mmol/L	6(66.7%)	7(24.1%)	1(25.0%)	11(35.5%)
	>1.7 mmol/L	3(33.3%)	22(75.9%)	3(75.0%)	20(64.5%)
MI type	Anterior	8(66.7%)	19(61.3%)	4(80.0%)	19(59.4%)
	Inferior	4(33.3%)	10(32.3%)	1(20.0%)	11(34.4%)
	Non ST Elevation	0(0.0)	2(6.5%)	0(0.0)	2(6.3%)

Normo-leptinemia: serum leptin level for male = (3.8 ± 1.8 ng/ml), and for female (7.4 ± 3.7 ng/ml) with BMI range 18–25.

Hyperleptinemia: serum leptin level > (3.8 ± 1.87 ng/ml) for males, for females > (7.4 ± 3.77 ng/ml) with BMI range 18–25.

Table 2 shows echocardiographic and coronary angiographic characteristics compared with mean and peak serum leptin sample.

**Table 2:** Echocardiographic & coronary angiographic characteristics compared with mean and peak leptin sample

**Variables**		**Mean Leptin**		**Peak Leptin**	
		**Normoleptinemia**	**Hyperleptinemia**	**Normoleptinemia**	**Hyperleptinemia**
		**N = 13**	**N = 31**	**N = 5**	**N = 33**
L. Atrium	1.9-4.0 cm	10(76.9%)	19(63.3%)	3(60.0%)	22(68.8%)
	>4.0 cm	3(23.1%)	11(36.7%)	2(40.0%)	10(31.3%)
LVEDD	≤5.6 cm	11(84.6%)	23(76.7%)	4(80.0%)	26(81.3%)
	>5.6 cm	2(15.4%)	7(23.3%)	1(20.0%)	6(18.8 %)
RVD	≤3.0 cm	12(92.3%)	30(100%)	5(100.0%)	31(96.9%)
	>3.0 cm	1(7.7%)	0(0.0)	0(0.0)	1(3.1%)
RVSP	≤30 mmHg	4(30.8%)	13(44.8%)	2(40.0%)	14(45.2%)
	>30 mmHg	9(69.25%)	16(55.2%)	3(60.0%)	17(54.8%)
FS%	≤25%	6(46.2%)	12(40.0%)	3(60.0%)	17(53.1%)
	>25%	7(53.8%)	18(60.0%)	2(40.0%)	15(46.9%)
EF%	≤45%	9(69.2)	16(53.3%)	1(20.0%)	13(40.6%)
	>45%	4(30.8%)	14(46.7%)	4(80.0%)	19(59.4%)
No of vessels	1VD	4(57.1%)	6(37.5%)	2(50.0%)	8(50.0%)
	2VD	3(42.9%)	6(37.5%)	2(50.0%)	4(25.0%)
	3VD	0(0.0)	6(37.5%)	0(0.0)	4(25.0%)
Recanalisation	Yes	7(87.5%)	15(71.4%)	4(80.0%)	15(75.0%)
(noninvasive assessment)	No	1(12.5%)	6(28.6%)	1(20.0%)	5(25.0%)

Normoleptinemia: serum leptin level for male = (3.8 ± 1.8 ng/ml), and for female (7.4 ± 3.7 ng/ml) with BMI range 18–25.

Hyperleptinemia: serum leptin level > (3.8 ± 1.87 ng/ml) for males, for females > (7.4 ± 3.77 ng/ml) with BMI range 18–25.

**Figure 1:**
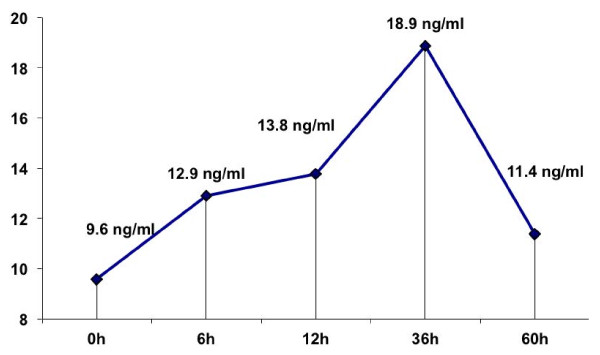
**Mean values of Serum leptin level (ng/ml) during the course of AMI at different time points (*P *value 0.04 based on repeated measures ANOVA).**

Figure 1 shows the mean values of serum leptin level during the course of AMI at different time points. Leptin concentrations (ng/ml) increased and peaked at fourth reading (36 hrs) after admission and gradually decreased thereafter (mean ± SD sample (1) =9.55 ± 7.4, sample (2) = 12.9 ± 8.4, sample (3) =13.8 ± 10.4, sample (4) =18.9 ± 18.1, sample (5) =11.4 ± 6.5, sample (6) =10.8 ± 8.9) ng/ml, *P* value 0.04 based on repeated measures ANOVA.

There was a significant correlation between serum leptin and BMI (r = 0.34; *p* = 0.03). Leptin levels also correlated significantly with serum creatine kinase level on the second day (r = 0.43, *p* ≤ 0.01). There was no difference in timing of peak serum levels between patients who had evidence of coronary reperfusion compared to those who did not achieve coronary reperfusion (*p* = 0.8), also there was no significant difference in mean serum leptin level in patients with anterior MI vs. inferior MI (mean ± SD = 11.71 ± 7, 9 vs.11.86 ± 6.6 ng/ml, *p* = 0.9) respectively. However, there was significant correlation with left ventricular ejection fraction, LVEF (r = −0.25, *p* = 0.01). No significant correlation between mean serum leptin and left atrial size (r = −0.09; *p* = 0.5), left ventricular systolic (r = 0.017, *p* = 0.9) or diastolic dimension (r = 0.1; *p* = 0.47) were found.

**Figure 2:**
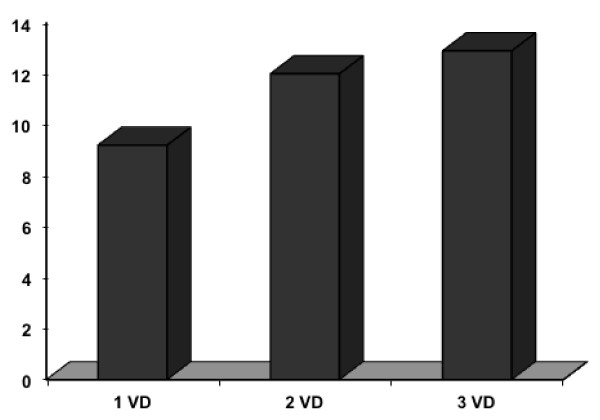
**Serum leptin level by number of diseased vessels (VD). (SVD = 9.2, 2VD = 12.0, 3VD = 12.9 ng/ml, *p*** **= 0.6).**

Figure 2 shows that mean serum leptin, which appeared to increase with the increase in the number of diseased vessels (mean, SVD = 9.2, 2VD = 12.0, 3VD = 12.9, *p* = 0.6), although this was not statistically significant.

Correlation between mean and peak leptin level and variables of interest showed significant correlation of mean serum leptin with the ejection fraction and body mass index (*P* < 0.05), and significant correlation of creatinine kinase with mean leptin level (*P* < 0.05) (Table 3).

**Table 3:** Correlation between mean Leptin level and variables of interest

	**Mean serum Liptin**	**Peak serum Liptin**
Lt. Atrium	−0.033	−0.061
LVEDD	−0.020	−0.094
LVESD	−0.128	−0.170
RVD	−0.146	−0.081
RVSP	−0.144	−0.145
FS%	0.189	0.126
EF%	0.306*	0.234
Mean CK	0.188	0.369*
BMI	0.287*	0.152
Mean CKMB	0.188	0.004
Age	0.122	0.038
Mean Troponin	−0.295	−0.277

*Two sided p value less than 0.05.

LVEDD: left ventricular diastolic dimension, LVESD: left ventricular systolic dimension, RVD: Right ventricular dimension, RVSP: right ventricular systolic pressure, FS: fraction shortening, EF: ejection fraction.

## Discussion

Studies on the role of serum leptin in coronary artery disease are scarce [24-34] (Table 4). To the best of our knowledge, this study is among few studies that analyzed the serial serum leptin levels in the setting of acute myocardial infarction and the first that evaluated its correlation with angiographic and echocardiographic findings in this high-risk group.

**Table 4:** Studies on leptin in acute myocardial infarction

**study**	**patients**	**conclusion**
Hadi Khafaji et al. 2011 Qatarcurrent study	87 pts: 51 AMI pts + 36 matched control	Leptin level is significantly correlated with BMI, CK, and LVEF. But neither with the type of MI (anterior vs. inferior) nor with the severity of angiographic findings and other echocardiographic parameters.
Yan GT,et al. * 2005 China [28]	AMI and CS pts	Leptin levels of both AMI & coronary atherosclerosis pts are significantly without a significant difference between each other, no correlation for leptin with CRP, TnT & endothelin
Krasnodebski et al. 2010 Poland [24]	58 patients with AMI	Plasma leptin levels in diabetic pts are significantly in acute stage of AMI than in the period of convalescence.
Wallander M,et al. 2008Sweden [29]	181 AMI	Circulating levels of leptin on the first morning after an AMI are associated with the presence of abnormal Glucose tolerance at discharge & with poor long-term prognosis.
Piestrzeniewicz et al. 2007 Poland [31]	40 obese and 40 non-obese men.	Leptinemia is associated with fasting glucose, triglyceride levels, CRP & uric acid & negatively with HDL-C; leptin may be a pathogenetic factor in cardiovascular disease
Taneli et al. 2006 Turkey [30]	35 pts stable angina + 40 STEMI, + 30 control	S. leptin is significantly in both stable angina &STEMI groups. No significant correlation between leptin levels & selected risk factors
Amasyali et al. 2006Turkey [25]	41 pts with AMI have thrombolysis	Failure of reperfusion therapy with streptokinase is significantly in patients with admission plasma leptin ≥14 ng/mL vs. pts with admission plasma leptin <14 ng/mL.
Selvakumar et al. 2005 India [32]	94 pts with acute ST EMI + 46 controls	leptin level is in patients with acute STEMI
Meisel et al. 2001 Isreal [26]	30 pts, AMI	Leptin peaked on the 2nd day of hospitalization with a 2-fold from its baseline level on admission (*p* < 0.02). On day 3, leptin levels, & were 46%, 9%, &6% above baseline on days 3, 4 and 5, respectively.
Fujimaki et al. 2001 Japan [35]	21 AMI pts 15 control	Hypoleptinaemic AMI pts had significantly plasma LDH vs. normoleptinaemic pts. No differences in other serum markers between normo vs. hyperleptinaemic AMI
Söderberg et al. 1999 Sweeden [33]	62 men with first-ever AMI	High leptin (OR 8.97; 95% CI: 1.73-46.5) & cholesterol (OR 5.18; 95% CI: 1.34-20.0) levels significant risk factors for AMI in a multivariate model
Stejskal et al. 1988 Czech** [34]	16 AMI, 22 unstable angina	Early after the acute coronary event, leptinemia in persons with AMI statistically positively correlated with concentration of interleukine-6 & subsequently of markers of coronary lesion severity (cTnI).

*Article in Chinese, information taken from the abstract, ** Article in Czech information taken from the abstract.

AMI: acute myocardial infarction, STEMI: STelevation myocardial infarction, cTnI): cardiac troponin I. BMI: Body mass index, CK: creatinine kinase, LVEF: left ventricular ejection fraction, HDL-C: high density lipoprotein cholesterol, LDH: lactate dehydrogenase, OR: odd rartio. CI confidence interval

In the United States population, increased leptin concentrations was significantly associated with increased risk of myocardial infarction and stroke in men and women, independent of traditional cardiovascular risk factors and obesity status [36]. We have shown previously a significant correlation between serum leptin and hs-CRP in stable cardiac patients [37]. Study by Stangl et al. [38] concluded that patients with coronary artery disease exhibited higher serum leptin concentrations than controls matched for age, gender & BMI, suggesting that leptin could contribute to the development of cardiovascular disease, possibly via activation of the sympathetic nervous system. The Trp64Arg variant of the β-adrenoceptor did not influence serum leptin levels [38]. Leptin might be a marker of risk of coronary artery disease, at least in men, and contributes to the risk profile in subjects with insulin resistance. Leptin concentrations were significantly higher in diabetic and coronary artery disease patients than in controls. Body weight, serum triglyceride concentration and systolic blood pressure were all significantly related to the logarithm of the serum leptin concentration in stable coronary artery disease patients [39]. Wallace et al. [40] documented that leptin is an independent risk factor for coronary artery disease using data from the west Scotland coronary prevention study. It has been suggests that leptin might participate in the catabolic state leading to development of cardiac cachexia in the course of congestive heart failure [41].

The current study demonstrates elevation of serum leptin levels in the acute phase of myocardial infarction and it peaks at 36 hours after admission (doubled). Whether leptin is released from the myocytes or it acts only as an acute phase reactant protein after its release from adipose tissue, was the main issue of this project. The fact that there was no earlier peak in serum leptin in patients who had coronary reperfusion after thrombolytic therapy compared to those who don’t achieve reperfusion suggests that leptin is not released from the myocytes and is mainly an acute phase reactant similar to high sensitivity C- reactive protein. This finding is concordant to the study in Poland, which included 35 patients with AMI and showed that plasma leptin levels in diabetic patients were significantly higher in AMI than in the period of convalescence. These findings suggest that leptin may play an important role in the metabolic changes taking place during the first days of AMI [24].

Correlating mean and peak serum leptin levels; serum leptin is positively correlated with the extent of diseased coronary vessels (1-, 2-, and 3 -vessel disease) (Figure 2), although statistically not significant, expanding the study sample may be confirmatory of this finding. Furthermore no significant correlation with either evidence of coronary re-canalization or with the type of MI (whether anterior or inferior) was found. We observed a significant correlation between serum leptin and LVEF with no significant correlation with other echocardiographic findings. Significant correlations were found between high serum leptin level and BMI as was demonstrated by previous studies (Table 3), with no significant correlation to other cardiovascular risk factor; diabetes, hypertension, total cholesterol LDL, HDL or triglyceride in this patients’ population. In contrast to our study which has followed the leptin up to 60 hours after the onset of AMI, investigators from Turkey studied the influence of plasma leptin concentrations obtained at the time of admission and 6 hours afterwards in 41 AMI patients who were treated with thrombolytic therapy. The investigators found that failure of reperfusion therapy with streptokinase was significantly higher in patients with admission plasma leptin concentrations ≥14 ng/mL as compared to patients with admission plasma leptin concentrations <14 ng/m, i.e. hyperleptinemia decreased the chance of successful reperfusion . Left ventricular ejection fraction was slightly but significantly higher in patients with admission plasma leptin concentrations ≥14 ng/mL than in patients with admission plasma leptin concentrations <14 ng/m (*p* = 0.031) [25]. Another study involving 30 consecutive AMI patients with a similar profile to the current one, showed that leptin levels reached its peak on the second day of hospitalization, with a 2-fold increase from baseline level on admission (*p* < 0.02). On day 3, leptin levels declined, and were 46%, 9%, and 6% above baseline on days 3, 4 and 5, respectively, suggesting that leptin may have a role in the metabolic changes taking place during the first days after an AMI. [26].

The predictive power of leptin on cardiovascular disease was addressed in a report from the Quebec cardiovascular study[27], eighty-six patients who developed ischemic heart disease were compared with referent matched for a number of traditional cardiovascular risk factor including body mass index. Leptin did not emerge as a predictor in coronary artery disease. However, fundamental differences between the two studies, first and most importantly, patients in our present study were all proven first ever AMI cases according to newly defined criteria [12], whereas in Quebec, the study group constituted a mixture of stable and unstable angina [27]. Furthermore Fujimaki et al. [35] correlated serum leptin with other myocardial infarction markers and interleukin level in 15 aged-matched controls and found a significant negative correlation between these two markers. These studies again suggest that leptin may play an important role in the metabolic changes taking place during the first days of myocardial infarction.

### Limitations of the study

Since this study included a small number of patients, the result should be interpreted cautiously.

## Conclusion

Serum leptin acts as an acute phase reactant in AMI patients. Significant correlation was found in mean serum leptin level with BMI, CK, and LVEF. There was no significant difference in mean serum leptin level in patients with anterior vs. inferior infarction and statistically no significant correlations of serum leptin with severity of angiographic findings and other echocardiographic parameters.

## Abbreviations

BMI: body mass index; SVD: single vessel disease; 2VD: two vessel disease; 3VD: three vessel disease; LVEF: left ventricular ejection fraction; RVD: right ventricular dimension; RVSP: right ventricular systolic pressure; STEMI: ST elevation myocardial infarction.

## Competing interests

The authors declare that they have no competing interests.

## Authors’ contributions

HARHK- participated in the design of the study, patients’ recruitment, writing, analyzing and reviewing the paper. AB- performed the statistical analysis. NR- participated in the design of the study and the performance of laboratory investigations. JA- participated in the design of the study and patients’ enrollment. All authors read and approved the final manuscript.

## References

[B1] Zhang Y Proenca R Maffei M Barone M Leopold L Friedman JM Positional cloning of the mouse obese gene and its human homologue Nature 1994 372 425 10.1038/372425a0 7984236

[B2] Halaas JL Gajiwala KS Maffei M Cohen SL Chait BT Rabinowitz D Lallone RL Burley SK Friedman JM Weight-reducing effects of the plasma protein encoded by the obese gene Science 1995 269 543 10.1126/science.7624777 7624777

[B3] Pelleymounter MA Cullen MJ Baker MB Hecht R Winters D Boone T Collins F Effects of the obese gene product on body weight regulation in ob/ob mice Science 1995 269 540 10.1126/science.7624776 7624776

[B4] Campfield LA Smith FJ Guisez Y Devos R Burn P Recombinant mouse ob protein: evidence for a peripheral signal linking adiposity and central neural networks Science 1995 269 546 10.1126/science.7624778 7624778

[B5] Sharma K Considine RV The Ob protein (leptin) and the kidney Kidney Int 1998 53 1483 10.1046/j.1523-1755.1998.00929.x 9607179

[B6] Sundell J Huuppon R Raitakari OT Nuutila P Knuuti J High serum leptin is associated with attenuated coronary vasoreactivity Obes Res 2003 11 6 776 782 10.1038/oby.2003.108 12805399

[B7] Matsuda K Teragawa H Fukuda Y Nakagawa K Higashi Y Chayama K Leptin causes nitric-oxide independent coronary artery vasodilation in humans Hypertens Res 2003 Feb 26 2 147 152 10.1291/hypres.26.147 12627874

[B8] Aizawa-Abe M Ogawa Y Masuzaki H Ebihara K Satoh N Iwai H Matsuoka N Hayashi T Hosoda K Inoue G Yoshimasa Y Pathophysiological role of leptin in obesity-related hypertension J Clin Invest 2000 105 1243 10.1172/JCI8341 10791999 PMC315441

[B9] Agata J Masuda A Takada M Higashiura K Murakami H Miyazaki Y Shimamoto K High plasma immunoreactive leptin level in essential hypertension Am J Hypertens 1997 10 1171 10.1016/S0895-7061(97)00310-5 9370390

[B10] Bodary PF Westrick RJ Wickenheiser KJ Shen Y Eitzman DT Effect of leptin on arterial thrombosis following vascular injury in mice JAMA 2002 287 1706 10.1001/jama.287.13.1706 11926895

[B11] Rizk NM Stammsen D Preibisch G Eckel J Leptin and tumor necrosis factor-alpha induce the tyrosine phosphorylation of signal transducer and activator of transcription proteins in the hypothalamus of normal rats in vivo Endocrinology 2001 Jul 142 7 3027 3032 10.1210/en.142.7.3027 11416024

[B12] Alpert JS Thygesen K Antman E Bassand JP Myocardial infarction redefined –a consensus document of the Joint European Society of Cardiology/American College of cardiology Committee for redefinition of of myocardial infarction J Am Coll Cardiol 2000 36 3 959 973 10.1016/S0735-1097(00)00804-4 10987628

[B13] Hadi HAR Al Suwaidi J, Bener A, Al Binali H: Thrombolytic therapy use in acute myocardial infaction and outcome in Qatar, *int J* Cardiol 2005 102 249 254 10.1016/j.ijcard.2004.05.024 15982492

[B14] Ma Z Gingerich RL Santiago JV Klein S Smith CH Landt M Radioimmunoassay of Leptin in Human Plasma Clin Chem June 1996 42 942 946 8665687

[B15] Fridewald WF Levy RI Frederickson DS Estimation of LDLcholesterol concentration without use of the Preparative Ultra-centrifuge Clin Chem 1972 18 499 502 4337382

[B16] Nauck M Warnick GR Rifai N Methods for measurement of LDL-cholesterol: a critical assessment of direct measurement by homogeneous assays versus calculation Clin Chem 2002 48 236 254 11805004

[B17] Panteghini M Pagani F Yeo KTJ, Apple FS, Christenson RH, Dati F, Mair J, Ravkilde J Wu AH: Committee on Standardization of Markers of Cardiac Damage of the IFCC. Evaluation of imprecision for cardiac troponin assays at low-range concentrations. Clin Chem 2004 50 327 332 10.1373/clinchem.2003.026815 14656904

[B18] Boden G Chen X Kolaczynski JW Polansky M Effects of prolonged hyperinsulinemia on serum leptin in normal human subjects J Clin Invest 1997 100 1107 10.1172/JCI119621 9276727 PMC508285

[B19] Licinio J Negrao AB Mantzoros C Kaklamani V Wong ML Bongiorno PB Mulla A Cearnal L Veldhuis JD Flier JS Synchronicity of frequently sampled, 24-h concentrations of circulating leptin, luteinizing hormone, and estradiol in healthy women Proc Natl Acad Sci U S A 1998 95 2541 10.1073/pnas.95.5.2541 9482922 PMC19406

[B20] Schoeller DA Cella LK Sinha MK Caro JF Entrainment of the diurnal rhythm of plasma leptin to meal timing J Clin Invest 1882 1997 100 10.1172/JCI119717 PMC508375 9312190

[B21] Rogers WJ Coggin CJ Gersh BJ Fisher LD Myers WO Oberman A Sheffield LT Ten-year follow-up of quality of life in patients randomized to receive medical therapy or coronary artery bypass graft surgery: The Coronary Artery Surgical Study (CASS) Circulation 1990 82 1647 10.1161/01.CIR.82.5.1647 1977531

[B22] Cerqueira MD Weissman NJ Dilsizian V Jacobs AK Kaul S Laskey WK Pennell DJ Rumberger JA Ryan T Verani MS American Heart Association Writing Group on Myocardial Segmentation and Registration for Cardiac Imaging. Standardized myocardial segmentation and nomenclature for tomographic imaging of the heart: a statement for healthcare professionals from the Cardiac Imaging Committee of the Council on Clinical Cardiology of the American Heart Association Circulation 2002 105 539 10.1161/hc0402.102975 11815441

[B23] Norusis MJ SPSS/PC + for windows. Base System and Advanced Statistical User’s Guide, Window Version Chicago, Illinois 1998 12

[B24] Krasnodebski P Bak MI Opolski G Karnafel W Leptin in acute myocardial infarction and period of convalescence in patients with type 2 diabetes mellitus Kardiol Pol 2010 Jun 68 6 648 653 20806194

[B25] Amasyali B Aytemir K Kose S Kilic A Abali G Iyisoy A Kursaklioglu H Turan M Bingol N Isik E Admission plasma leptin level strongly correlates with the success of thrombolytic therapy in patients with acute myocardial infarction Angiology 2006 57 6 671 680 17235106 10.1177/0003319706295204

[B26] Meisel SR Ellis M Pariente C Pauzner H Liebowitz M David D Shimon I Serum leptin levels increase following acute myocardial infarction Cardiology 2001 95 4 206 211 10.1159/000047373 11585996

[B27] Després JP Lupien PJ Moorjani S Dagenais GR Cantin B Mauriege P Lamarche B Couillard C Leptenemia is not a risk factor for ischemic heart disease in men. Prospective results from Quebec Cardiovascular study Diabetic Care 1998 21 782 786 10.2337/diacare.21.5.782 9589240

[B28] Yan GT Xue H Lin J Hao XH Zhang K Wang LH Correlation analysis of increase in serum level of leptin with that of C reactive protein, troponin T and endothelin in patients with acute myocardial infarction Zhongguo Wei Zhong Bing Ji Jiu Yi Xue 2005 Sep 17 9 530 532 16146596

[B29] Wallander M Söderberg S Norhammar A Leptin: a predictor of abnormal glucose tolerance and prognosis in patients with myocardial infarction and without previously known Type 2 diabetes Diabet Med 2008 Aug 25 8 949 955 10.1111/j.1464-5491.2008.02509.x 18959608

[B30] Taneli F Yegane S Ulman C Tikiz H Bilge AR Ari Z Uyanik BS Increased serum leptin concentrations in patients with chronic stable angina pectoris and ST-elevated myocardial infarction Angiology 2006 57 3 267 272 10.1177/000331970605700302 16703186

[B31] Piestrzeniewicz K Luczak K Komorowski J Maciejewski M Goch JH The relationship between leptin and obesity and cardiovascular risk factors in men with acute myocardial infarction Cardiol J 2007 14 3 252 259 18651469

[B32] Selvakumar D Selvakumar PV George P Jose VJ Mariappan P Serum leptin levels in acute myocardial infarction Indian Heart J 2005 57 1 39 43 15852893

[B33] Söderberg S Ahrén B Jansson JH Johnson O Hallmans G Asplund K Olsson T Leptin is associated with increased risk of myocardial infarction J Intern Med 1999 Oct 246 4 409 418 10.1046/j.1365-2796.1999.00571.x 10583712

[B34] Stejskal D Růzicka V Bartek J Horalík D Leptinemia in persons with acute myocardial infarct Vnitr Lek 1998 Oct 44 10 588 592 10422492

[B35] Fujimaki S Kanda T Fujita K Tamura J Kobayashi I The significance of measuring plasma leptin in acute myocardial infarction J Int Med Res 2001 29 2 108 113 11393343 10.1177/147323000102900207

[B36] Sierra-Johnson J Romero-Corral A Lopez-Jimenez F Gami AS Sert Kuniyoshi FH Wolk R Somers VK Relation of increased leptin concentrations to history of myocardial infarction and stroke in the United States population Am J Cardiol 2007 100 2 234 239 10.1016/j.amjcard.2007.02.088 17631076 PMC2000836

[B37] Khafaji HAR Hadi Bener A Osman M Al-marri A Al Suwaidi J The impact of diurnal fasting during Ramadan on the lipid profile, hs-CRP, and serum leptin in stable cardiac patients Vascular Health and Risk Management 2012 8 7 14 22272070 10.2147/VHRM.S22894 PMC3262481

[B38] Stangl K Cascorbi I Laule M Stangl V Vogt M Ziemer S Roots I Wernecke K Baumann G Hauner H Elevated serum leptin in patients with coronary artery disease: no association with the Trp64Arg polymorphism of the beta3-adrenergic receptor Int J Obes Relat Metab Disord 2000 Mar 24 3 369 375 10.1038/sj.ijo.0801159 10757633

[B39] Al-Daghri N Al-Rubean K Bartlett WA Al-Attas O Jones AF Kumar S Serum leptin is elevated in Saudi Arabian patients with metabolic syndrome and coronary artery disease Diabet Med 2003 Oct 20 10 832 837 10.1046/j.1464-5491.2003.01044.x 14510865

[B40] Wallace AM Mc Mahan AD Packed CJ Kelly A Shepherd J Gaw A Sattar N Plasma Leptin and the risk of cardiovascular disease in the wast of Scotland coronary prevention study Circulation 2001 104 3052 3056 10.1161/hc5001.101061 11748099

[B41] Schuler G Moebius-Winkler S Erbs S Gielen S Adams V Schoene N Linke A Kratzsch J Schule PC Elevated serum levels of leptin and soluble leptin receptor in patients with advanced heart failure EurJ Heart Fail 2003 33 1 10.1016/s1388-9842(02)00177-0 12559213

